# AlNiCo Magnet with NdFeB-Nanocrystalline Phase Prepared by Spark Plasma Sintering

**DOI:** 10.3390/ma18081847

**Published:** 2025-04-17

**Authors:** Haifeng Lan, Yueqing Liu, Jiangtao Zhao, Lei Liu, Xiaoqiang Yu, Tianyu Hu, Yingli Sun, Yong Ding, Aru Yan

**Affiliations:** 1 Jiangxi Provincial Key Laboratory of Magnetic Metallic Materials and Devices, College of Rare Earths, Jiangxi University of Science and Technology, Ganzhou 341000, China; 2 CISRI & NIMTE Joint Innovation Center for Rare Earth Permanent Magnets, Ningbo Institute of Materials Technology and Engineering, Chinese Academy of Sciences, Ningbo 315201, China; 3 National Rare Earth Funetional Material Innovation Center, Ganzhou 341000, China

**Keywords:** spark plasma sintering, magnetocrystalline anisotropy, nanocrystalline, AlNiCo magnet

## Abstract

Magnetocrystalline anisotropy has many advantages over shape anisotropy regarding coercivity in permanent magnets, making it a promising approach to enhance the coercivity of AlNiCo magnets. In this work, AlNiCo magnets with NdFeB-nanocrystalline phase were prepared by spark plasma sintering (SPS), and the effect of the NdFeB phase on coercivity was uncovered. AlNiCo powder with a spinodal structure and NdFeB powder with a nanocrystalline structure, which exhibit shape anisotropy and magnetocrystalline anisotropy, respectively, were sintered by SPS. With the advantages of low-temperature densification achieved by the SPS process, the spinodal and nanocrystalline structures were mostly retained. The microstructure analysis indicated that the SPS-ed magnet primarily consisted of AlNiCo regions with a spinodal structure, NdFeB regions with a nanocrystalline structure, and a transition region approximately 1~7 µm wide between them. A significant effect of the magnetic anisotropy of the NdFeB phase on magnetization behavior was found. The hysteresis loop of the SPS-ed magnets became single-phase magnetization, in contrast with the double-phase magnetization observed in the simple mixed powder. As the magnetocrystalline anisotropy of the NdFeB phase possesses higher coercivity, the coercivity of the SPS-ed magnet increased from 1250 Oe (of the AlNiCo raw powder) to 2490 Oe. This work provides valuable information for the coercivity enhancement of AlNiCo magnets.

## 1. Introduction

AlNiCo magnets play an irreplaceable role in high-temperature applications and precision instrumentation due to their high Curie temperature and excellent magnetic stability [1,2]. However, the low coercivity, which leads to low maximum energy product, restricts the applications of AlNiCo magnets. Consequently, enhancing coercivity has become a primary focus of the current research on AlNiCo magnets [3,4,5]. AlNiCo magnets have a spinodal structure, which is formed by spinodal decomposition. The magnetic FeCo-rich (α_1_) phase and the AlNi-rich (α_2_) phase are arranged periodically. The coercivity of AlNiCo magnets primarily depends on the shape anisotropy of the spinodal structure [6,7,8,9]. To advance the shape anisotropy field, previous studies have optimized the magnet composition [10,11,12] or refined the heat treatment processes [13,14]. For instance, Ahmad et al. [10] adjusted the content of the Co and Nb elements, resulting in a coercivity increase to approximately 1900 Oe for an AlNiCo alloy. Zhou et al. [13] optimized the temperature and holding time of a magnetic field heat treatment, which also improved coercivity. However, coercivity remained consistently below 2000 Oe due to the intrinsic limitation of the shape anisotropy [6,15]. Magnetocrystalline anisotropy is a crystalline structure that shows distinct magnetic properties along different axial directions. The coercivity controlled by magnetocrystalline anisotropy is tens of times higher than that controlled by shape anisotropy [15]. Based on this, RECo-type, REFeB-type, and other magnets (RE: rare earth) have been developed. Recent studies have introduced the magnetocrystalline anisotropic phase (Y_2_Co_17_ phase and Dy_2_Co_17_ phase) into AlNiCo magnets by doping RE elements during casting, slightly increasing the coercivity of AlNiCo magnets [16,17]. However, the magnetocrystalline anisotropic phase can only be formed by the thermodynamic equilibrium of the alloy and, at the same time, it causes the destruction of the spinodal structure in AlNiCo magnets. Spark plasma sintering (SPS) technology is one of the most advanced rapid hot-pressing sintering technologies [18,19,20]. It allows powders to be quickly densified at low temperatures while avoiding excessive grain growth [21,22]. Recent studies have successfully prepared nanocrystalline NdFeB magnets using SPS, achieving 99% densification at a sintering temperature of 700 °C with a grain size of approximately 200 nm [23,24]. In this paper, AlNiCo magnets with an NdFeB-nanocrystalline phase are prepared by SPS. The effect of the NdFeB phase on the coercivity of the magnet is systematically investigated.

## 2. Materials and Methods

**Figure 1:**
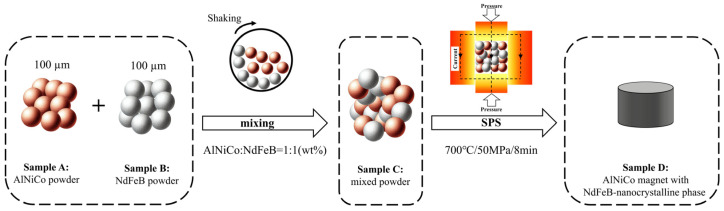
Schematic workflow for the preparation of AlNiCo magnets with NdFeB-nanocrystalline phase.

To prepare AlNiCo magnets with an NdFeB-nanocrystalline phase, we used AlNiCo powder and NdFeB powder, both with an average particle size of approximately 100 µm. The AlNiCo powder was derived from commercial AlNiCo 8 magnets with a nominal composition of 34Fe-7Al-14Ni-36Co-6Ti-3Cu (wt%), while the NdFeB powder was derived from commercial MQU-F powders with a nominal composition of 30.5Nd-64.1Fe-4.0Co-0.5Ga-0.9B (wt%). Figure 1 shows the schematic workflow for the preparation of AlNiCo magnets with an NdFeB-nanocrystalline phase. The AlNiCo and NdFeB powders were placed in an airtight container under Ar atmosphere at a ratio (AlNiCo: NdFeB) of 1:1 (wt%) and shaken for 1h to obtain the mixed powder. Prolonged shaking was performed to ensure uniformity of the mixed powder. The mixed powder was sintered by spark plasma sintering (SPS, LANBOX-1575F, SINTER LAND INC., Niigata, Japan) at 700 °C for 8 min under a constant pressure of 50 MPa, and the vacuum value of the chamber was less than 10^−3^ Pa during the sintering process. These SPS conditions were optimized in our SPS facility. The AlNiCo powder, NdFeB powder, mixed powder, and AlNiCo magnet with an NdFeB nanocrystalline phase were named A, B, C, and D, separately.

The density was determined by employing Archimedes’ principle (drainage method). The phase composition was analyzed using X-ray diffraction (XRD, D8 Advance, Bruker, Karlsruh, Germany) with Cu Kα radiation and differential scanning calorimetry (DSC, 404F1, NETZSCH, Selb, Germany). The microstructure and elemental distribution were analyzed using scanning electron microscopy (SEM, G300, ZEISS, Oberkochen, Germany), along with an energy dispersive spectrometer (EDS), electron probe micro-analysis (EPMA, JXA-iHP200F, JEOL, Musashino, Japan), and transmission electron microscopy (TEM, Talos F200X, Themo Fisher, Waltham, MA, USA). The TEM samples were prepared by the focused ion beam (FIB) technique, with the samples selectively extracted from specific areas. The magnetic hysteresis loops of the magnets were measured using a physical property measurement system (PPMS, DynaCool, Quantum Design, San Diego, CA, USA) equipped with a vibrating sample magnetometer (VSM).

## 3. Results and Discussion

**Figure 2:**
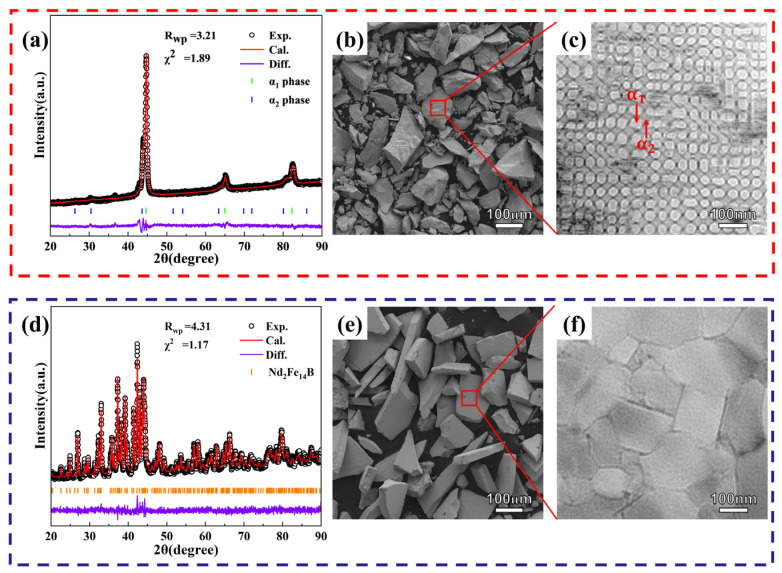
XRD patterns of (**a**) sample A, (**d**) sample B; SEM images of (**b**) sample A, (**e**) sample B; and TEM images of (**c**) sample A, (**f**) sample B.

To ensure that sample A exhibited a spinodal structure with shape anisotropy and sample B possessed a nanocrystalline structure with magnetocrystalline anisotropy, the phase compositions of both samples were analyzed. In Figure 2a, the XRD patterns demonstrate that sample A is composed of the FeCo-type α_1_ phase (PDF#65-7519) and the AlNi_2_Ti-type α_2_ phase (PDF#65-4198). Figure 2b shows the SEM image of sample A. Sample A primarily consists of bulk particles in the form of cuboids, with a few fragments present. The average particle size of sample A is 76.9 ± 21.2 µm. The TEM image of sample A is shown in Figure 2c. The α_1_ rounds with an average size of approximately 30 nm are periodically embedded in the α_2_ phase, exhibiting a checkerboard-like morphology. The nanostructure of sample A displays the spinodal structure of typical AlNiCo magnets [6]. Adopting the similar scheme, sample B is composed of an Nd_2_Fe_14_B phase (PDF#39-0473). The average particle size of sample B is 83.1 ± 21.3 µm. The nanostructure of sample B consists of equiaxed crystals with a grain size ranging from 50 to 100 nm, which is characteristic of a typical nanocrystalline structure [25].

**Figure 3:**
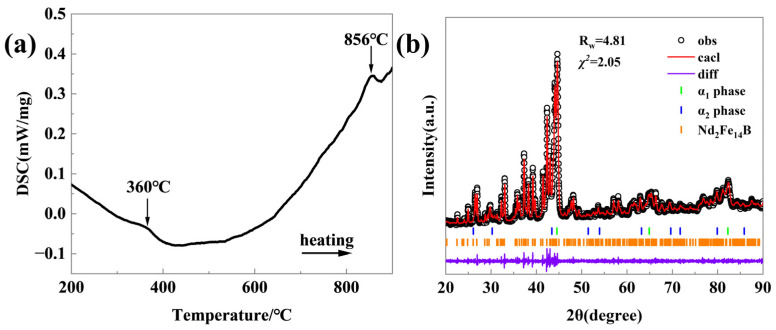
(**a**) DSC curve and (**b**) XRD pattern of sample D.

Sample A with a spinodal structure and sample B with a nanocrystalline structure were mixed and underwent SPS. To achieve densification of the SPS-ed sample while retaining the NdFeB-nanocrystalline phase, the process parameters including composition ratio, SPS temperature, and holding time were optimized systematically. Under the process parameters of AlNiCo/NdFeB = 1:1 (wt%), an SPS temperature of 700 °C, and a holding time of 8 min, an SPS-ed sample (sample D) with a density of 6.9 g/cm^3^ and a relative density of 92% was obtained. The phase composition, microstructure, and nanostructure of sample D were systematically analyzed. The phase composition was characterized by DSC and XRD. The DSC testing temperature range was from room temperature to 900 °C, with a constant heating rate of 20 °C/min. The Ar was continuously filled at a flow rate of 50 mL/min during measuring. In Figure 3a, the DSC curve exhibits two endothermic peaks at 360 °C and 856 °C, respectively. Combining the raw material composition, the peak at 360 °C corresponds to the NdFeB phase, while the peak at 856 °C corresponds to the AlNiCo phase. To further determine the phase composition proportions, the XRD pattern was analyzed using the Jade 6 software, and Rietveld refinement was conducted using the GSAS Ⅱ software. In Figure 3b, the XRD pattern indicates that sample D is composed of the α_1_ phase, α_2_ phase, and Nd_2_Fe_14_B phase. Table 1 shows the lattice parameters and content of α_1_ phase, α_2_ phase and Nd_2_Fe_14_B phase calculated by Rietveld analysis. The contents of each phase are 26% α_1_ phase, 19.9% α_2_ phase, and 54.1% Nd_2_Fe_14_B phase. The ratio of the combined α_1_ phase and α_2_ phase content to the Nd_2_Fe_14_B phase content is approximately 1:1, which is similar to the raw material ratio.

**Figure 4:**
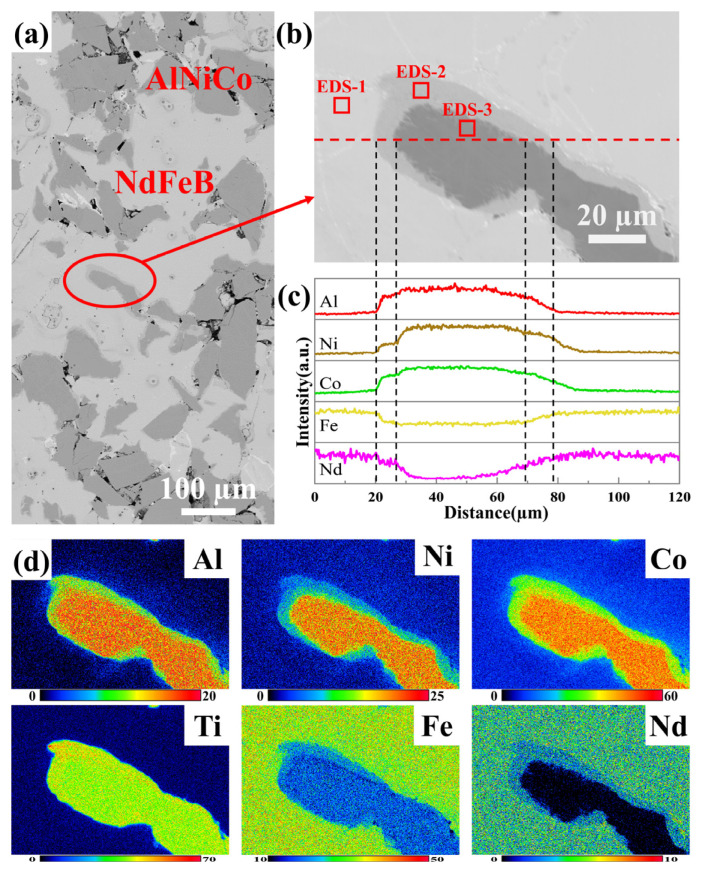
SEM image of sample D. Observe the vertical pressure direction in SPS. (**a**) Low magnification; (**b**) high magnification (marked by the red ovals); (**c**) EDS results of red line in (**b**); (**d**) EPMA mapping of Al, Ni, Co, Ti, Fe, Nd elements in (**b**).

The microstructure of sample D was investigated by SEM and the corresponding EDS. The SEM image is shown in Figure 4. Sample D consists of the following three regions: a dark region, a light region, and a grey region. The dark and light regions are primary regions. The dark region is sparsely distributed in the light region, with an approximate width of 100 µm. The grey region, spanning 1~7 µm in width and situated between the two primary regions, is designated as transition region. According to the EDS analysis, the darker region has higher Al, Ni, and Co contents, designated as the AlNiCo region, whereas the brighter region is characterized by higher Nd and Fe contents, designated as the NdFeB region. The composition of three regions are listed in Table 2. The composition of the AlNiCo and NdFeB regions is similar to that of the raw materials. The transition region encompasses all the elements present in both the AlNiCo and NdFeB regions. The distribution of the Al, Ni, Co, Ti, Fe, and Nd elements is visually represented in the EPMA images.

**Figure 5:**
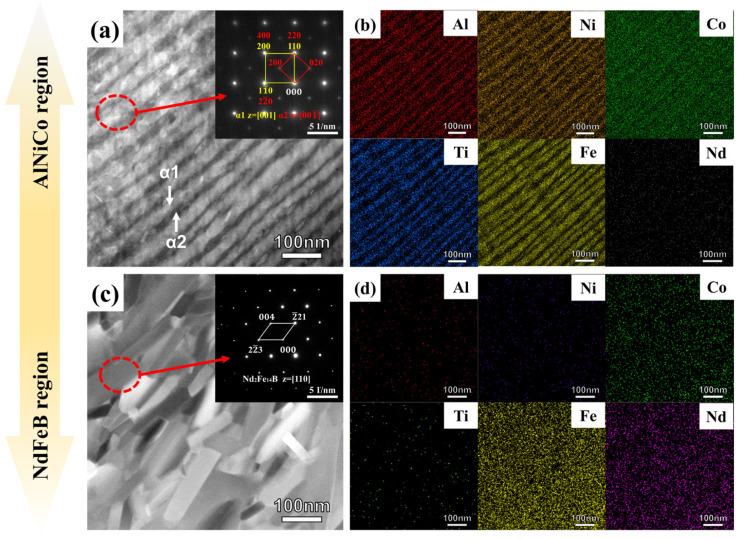
(**a**) HAADF STEM images and SAED pattern(inset) of AlNiCo region; (**b**) EDS element mapping of (**a**); (**c**) HAADF STEM images and SAED pattern (inset) of NdFeB region; (**d**) EDS element mapping of (**c**).

A further examination of the nanostructure in the AlNiCo region and NdFeB region was conducted using TEM and EDS analyses. The samples were observed in the direction perpendicular to the pressing direction during the SPS. The AlNiCo and NdFeB regions at various locations were observed to eliminate potential randomness in the observation area. The AlNiCo and NdFeB regions exhibited comparable morphological features regardless of their locations. Figure 5a displays the AlNiCo region. It presents two different rods that are alternately arranged. Each rod has a width of approximately 20 to 30 nm and a length of over 100 nm. The SADE pattern presented in Figure 5a shows that the two rods belong to the BCC structure. Combining the elemental distributions as shown in Figure 5b, the brighter rods are enriched in Fe and Co, determined as the α_1_ phase; while the darker rods are enriched in Al and Ni, determined as the α_2_ phase. The quantitative elemental content of α_1_ phase and α_2_ phase are shown in Table 3. The AlNiCo region exhibits the typical spinodal structure. The nanostructure and element distribution of the NdFeB region are illustrated in Figure 5c and Figure 5d, respectively. The NdFeB region is characterized by the presence of nanograins that exhibit a brick-like morphology, with lengths ranging from 90 to 150 nm, widths between 40 and 80 nm, and length-to-width ratios varying from 1.2 to 2. The NdFeB region exhibits a nanocrystalline structure. The SADE pattern presented in Figure 5c suggests that the nanocrystalline structure belong to the Nd_2_Fe_14_B phase, which is a magnetocrystalline anisotropy phase. The element contents of nanocrystalline, as shown in Table 3, is similar to that of the NdFeB powder. 

**Figure 6:**
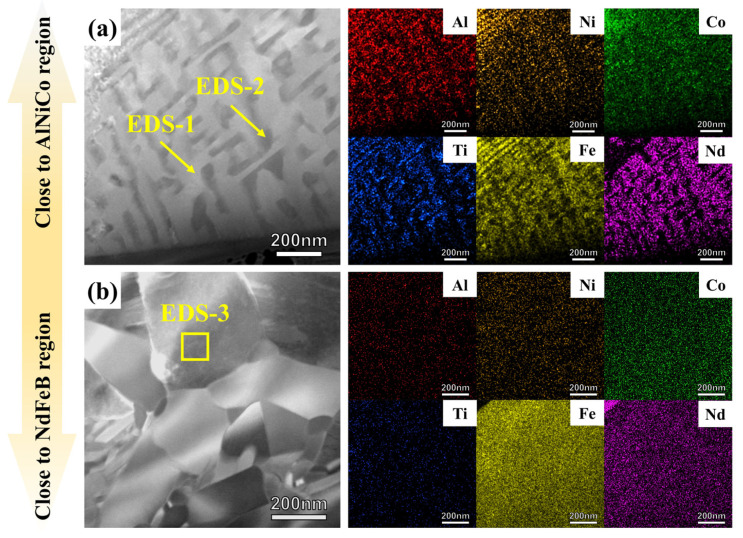
Morphology and elemental distribution in different regions of the transition region: (**a**) transition region close to AlNiCo region; (**b**) transition region close to NdFeB region.

The nanostructure characteristics of the transition region within the AlNiCo and NdFeB regions were further analyzed by TEM and EDS. The TEM image of transition region is shown in Figure 6. There are two different morphologies in the transition region. The transition region close to the AlNiCo region still consists of the Fe-rich phase and the Al-rich phase, as shown in Figure 6a. The dimensions of the Fe-rich and Al-rich phases have approximately doubled as compared to the rods within the AlNiCo region. The quantitative elemental content of Fe-rich phase and Al-rich phase are shown in Table 4. Nd elements are incorporated into the Fe-rich phase, while the Co elements diffuse from the Fe-rich phase into the Al-rich phase. The transition region close to the NdFeB region consists of coarse equiaxed grains, with a grain size of approximately 200 nm. In contrast with the nanocrystallines within the NdFeB region, the grain size grows by more than two times, which is presented in Figure 6b. The compositions of coarse equiaxed grains is shown in Table 4. Smaller radius atoms, such as Al and Ti, are incorporated into the nanocrystallines. The original spinodal structure and original nanocrystalline structure are disrupted.

**Figure 7:**
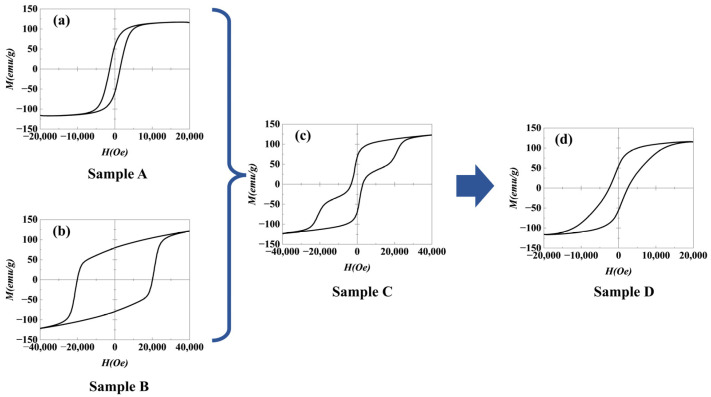
Hysteresis loops of different samples: (**a**) sample A; (**b**) sample B; (**c**) sample C; (**d**) sample D.

**Figure 8:**
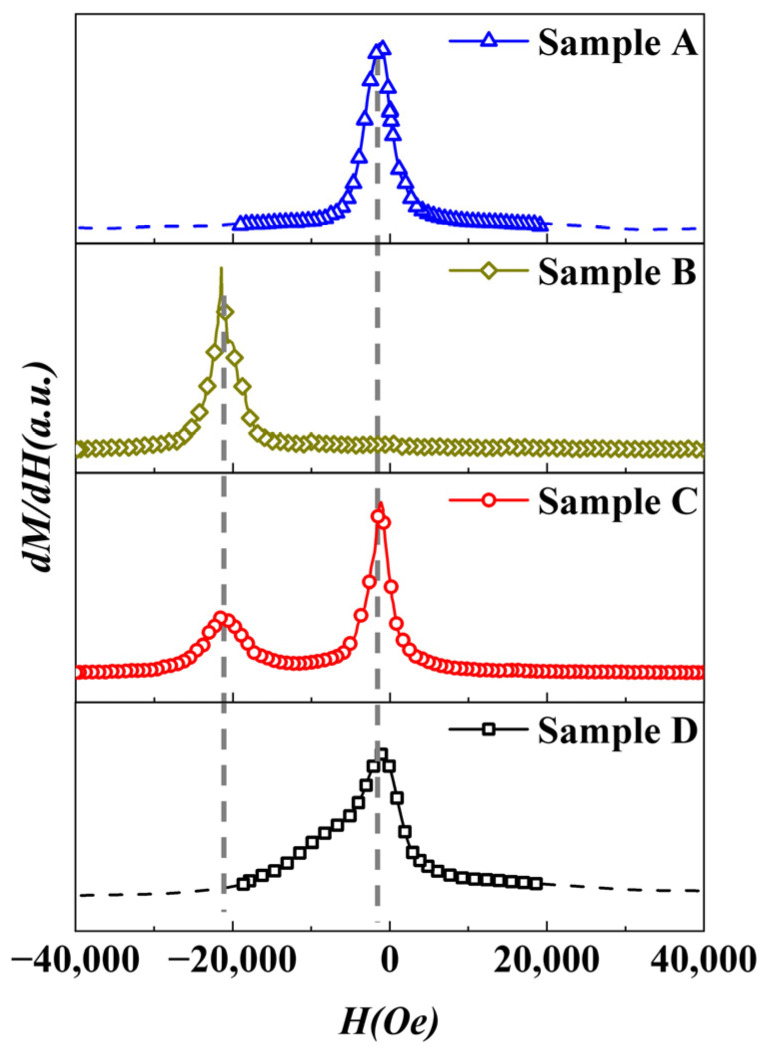
*dM/dH-H* curves of sample A, sample B, sample C, and sample D.

Compared to the casting method [16,17], the SPS method can effectively introduce the NdFeB-nanocrystalline phase while achieving densification at low temperatures. The AlNiCo magnets with an NdFeB-nanocrystalline phase were prepared, in which the microstructure consisted mainly of AlNiCo regions with a spinodal structure, NdFeB regions with a nanocrystalline structure, and a transition region approximately 1~7 µm wide between them. In order to investigate the effect of the NdFeB-nanocrystalline phase on the coercivity of an AlNiCo magnet, the magnetization behavior of sample D was analyzed. The hysteresis loops of different samples were measured by PPMS, as shown in Figure 7. The magnetic parameters of different samples, as obtained from Figure 7, are listed in Table 5. The demagnetization curves of different samples were derived, and the corresponding *dM/dH-H* curves are shown in Figure 8. Sample A and sample B both exhibit smooth hysteresis loops with single sharp peaks in their corresponding *dM/dH-H* curves (at approximately 1250 Oe and 21,000 Oe, respectively), which are characteristic of single-phase magnetization. In sample C, a pronounced step is observed in the hysteresis loop, and the *dM/dH-H* curve exhibits two distinct peaks corresponding to the peaks observed for sample A and sample B, respectively. The peak position and peak width both exhibited smaller changes compared to sample A and sample B. Sample C shows the typical double-phase magnetization behavior. For sample D, the hysteresis loop becomes smooth, and the corresponding *dM/dH-H* curve exhibits only a single peak (~1250 Oe). The peak width at high fields increases as compared to sample A, showing single-phase magnetization behavior. These indicate that the coupling effect of the NdFeB-nanocrystalline phase with the AlNiCo spinodal structure phase is excellent.

**Figure 9:**
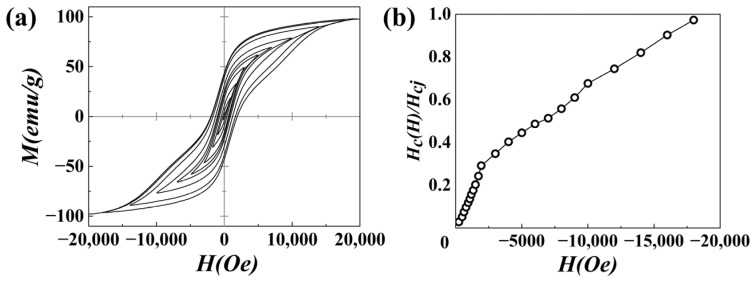
(**a**) Minor hysteresis loops; (**b**) dependence of coercivity on the field of sample D.

To further understand the role of the NdFeB-nanocrystalline phase, the minor hysteresis loop of sample D is measured, as shown in Figure 9a. The hysteresis loop increases with the increase in the external field. The dependence of coercivity on the field is shown in Figure 9b. It shows that the coercivity increases monotonically with the increase in the field, but there is a break point at about 2500 Oe. As the microstructure of sample D primarily consists of AlNiCo spinodal structure phase and the NdFeB-nanocrystalline phase, it is easy to understand that the AlNiCo spinodal structure phase is magnetized at low field and the NdFeB-nanocrystalline phase is magnetized at high field. In a low magnetic field, magnetization mainly occurs in the AlNiCo spinodal structure phase, and the coercivity increases rapidly with the increase in the external field. With the increase in the external field, the magnetized volume content of the NdFeB-nanocrystalline phase gradually increases, the significance of the NdFeB phase becomes increasingly prominent, and the coercivity continues to increase. Considering the magnetic coupling of the NdFeB-nanocrystalline phase, the increase in coercivity could be mainly attributed to the magnetic coupling effect of the NdFeB-nanocrystalline phase with the AlNiCo spinodal structure phase. Benefiting from the high coercivity and excellent coupling effect of the NdFeB-nanocrystalline phase, the coercivity of the AlNiCo magnet with an NdFeB-nanocrystalline phase increased significantly to 2490 Oe, compared to 1250 Oe for the AlNiCo powder.

## 4. Conclusions

This study aimed to prepare AlNiCo magnets with an NdFeB-nanocrystalline phase by spark plasma sintering (SPS), as well as to investigate the effect of the NdFeB phase on the coercivity of a magnet. The main findings are as follows:

1. AlNiCo powder with a spinodal structure and NdFeB powder with a nanocrystalline structure, which exhibit shape anisotropy and magnetocrystalline anisotropy, respectively, are sintered by SPS. The AlNiCo magnet with an NdFeB-nanocrystalline phase is prepared, in which the microstructure consists mainly of AlNiCo regions with a spinodal structure, NdFeB regions with a nanocrystalline structure, and an unavoidable transition region approximately 1~7 µm wide between them.
2. The hysteresis loop of the AlNiCo magnet with the NdFeB-nanocrystalline phase shows single-phase magnetization behavior, in contrast with the double-phase magnetization observed in simple mixed powders. With the high coercivity and excellent coupling effect of the NdFeB-nanocrystalline phase, the coercivity of the AlNiCo magnet with the NdFeB-nanocrystalline phase increased from 1250 Oe of the AlNiCo powder to 2490 Oe.

This work demonstrates an effective method for introducing a controllable magnetocrystalline anisotropy phase into AlNiCo magnets. The significant enhancement in coercivity provides valuable insights and inspiration for future research to improve the performance of AlNiCo magnets.

## Figures and Tables

**Table 1:** Lattice constants and content of α_1_ phase, α_2_ phase and Nd_2_Fe_14_B phase from Figure 3b.

Phase	Parameters			
	a (Å)	c (Å)	Volume (Å^3^)	Content (wt%)
α_1_	2.78	2.78	21.5	26.0
α_2_	6.02	6.02	218.5	19.9
Nd_2_Fe_14_B	8.5	12.6	115.0	54.1

**Table 2:** EDS results (wt%) in Figure 4b.

	Al	Ni	Co	Ti	Cu	Fe	Nd
EDS-1	-	-	6.5	-	-	65.6	27.8
EDS-2	2.3	6.0	27.6	4.3	1.9	37.7	20.3
EDS-3	6.2	13.1	37.9	5.3	2.8	34.7	-

**Table 3:** Phase compositions of the α_1_, α_2_, and Nd_2_Fe_14_B phases in sample D (wt%).

Phase	Al	Ni	Co	Ti	Cu	Fe	Nd
α_1_	2.5	7.6	38.6	1.6	1.7	48.0	-
α_2_	10.1	25.2	34.9	8.9	4.1	16.8	-
Nd_2_Fe_14_B	-	-	6.4	-	-	69.5	24.1

**Table 4:** EDS results in Figure 6 (wt%).

	Al	Ni	Co	Ti	Cu	Fe	Nd
EDS-1	1.7	3.2	20.8	1.5	1.0	50.9	20.7
EDS-2	6.8	6.6	45.6	16.6	0.5	19.4	4.6
EDS-3	0.6	-	9.2	0.2	-	68.5	21.5

**Table 5:** Magnetic parameters of different samples.

Sample	*H_c_* (Oe)	*M_r_* (emu/g)	*M_s_* (emu/g)
A	1250	39	110
B	21,000	79	123
C	3250	67	119
D	2490	55	116

## Data Availability

The original contributions presented in this study are included in the article. Further inquiries can be directed to the corresponding authors.

## Abbreviations

The following abbreviations are used in this manuscript:

*SPS*	spark plasma sinter
*H*	magnetic field
*M*	moment
*H_c_*	coercivity
*M_r_*	remanent magnetism
*M_s_*	saturation magnetism
